# Automated acute skin toxicity scoring in a mouse model through deep learning

**DOI:** 10.1007/s00411-024-01096-x

**Published:** 2024-11-06

**Authors:** Morten Sahlertz, Line Kristensen, Brita Singers Sørensen, Per Rugaard Poulsen, Folefac Charlemagne Asonganyi, Priyanshu Sinha, Jasper Nijkamp

**Affiliations:** 1 Danish Centre for Particle Therapy, Aarhus University Hospital, Aarhus, Denmark; 2 Department of Clinical Medicine, Aarhus University, Aarhus, Denmark; 3 Department of Oncology, Aarhus University Hospital, Aarhus, Denmark

**Keywords:** Skin toxicity, Deep learning, Preclinical radiotherapy, Observer study, Object detection, Classification

## Abstract

This study presents a novel approach to skin toxicity assessment in preclinical radiotherapy trials through an advanced imaging setup and deep learning. Skin reactions, commonly associated with undesirable side effects in radiotherapy, were meticulously evaluated in 160 mice across four studies. A comprehensive dataset containing 7542 images was derived from proton/electron trials with matched manual scoring of the acute toxicity on the right hind leg, which was the target area irradiated in the trials. This dataset was the foundation for the subsequent model training. The two-step deep learning framework incorporated an object detection model for hind leg detection and a classification model for toxicity classification. An observer study involving five experts and the deep learning model, was conducted to analyze the retrospective capabilities and inter-observer variations. The results revealed that the hind leg object detection model exhibited a robust performance, achieving an accuracy of almost 99%. Subsequently, the classification model demonstrated an overall accuracy of about 85%, revealing nuanced challenges in specific toxicity grades. The observer study highlighted high inter-observer agreement and showcased the model’s superiority in accuracy and misclassification distance. In conclusion, this study signifies an advancement in objective and reproducible skin toxicity assessment. The imaging and deep learning system not only allows for retrospective toxicity scoring, but also presents a potential for minimizing inter-observer variation and evaluation times, addressing critical gaps in manual scoring methodologies. Future recommendations include refining the system through an expanded training dataset, paving the way for its deployment in preclinical research and radiotherapy trials.

**Supplementary Information:**

The online version contains supplementary material available at 10.1007/s00411-024-01096-x.

## Introduction

Radiotherapy is a widely used treatment modality for effectively targeting and eradicating cancer cells. Nevertheless, the balance between tumor control and minimizing treatment-related morbidity is complex. Tumor control is the foremost objective, aiming to eradicate cancerous cells and tumors. Exposure of normal tissues to radiation can cause toxicities, in the case of skin, this can lead to erythema, desquamation and pain (Singh et al. 2016; Wei et al. 2019). Acute skin dermatitis can also negatively impact patients’ quality of life, with common quality of life issues such as decreased self-esteem, embarrassment, pain and impaired ability to complete mundane daily activities (Behroozian et al. 2023). To assess the severity of these skin reactions, researchers and clinicians have traditionally relied on manual scoring systems. These systems are subjective and prone to interobserver variability, limiting their accuracy and reliability (Fuzissaki et al. 2019; Partl et al. 2019).

In preclinical research, animal models play a vital role in understanding the intricate dynamics between tumor control and morbidity such as radiation-induced skin toxicities (Inada et al. 1980; Sørensen et al. 2022; Vozenin et al. 2019). Murine skin resembles human skin in many areas, making it an appropriate surrogate for investigating acute skin responses (Zomer and Trentin 2018). Since the 1970s, a range of radiobiological studies have used a model system, where mice are irradiated on one hind leg and the acute and late toxicities can be studied under varies conditions. The used in-house mouse model, as described in Overgaard C (2023)(Overgaard et al. 2023) is based on a slightly modified analysis table, that originated from Von der Maase (von der Maase 1984). However, the manual scoring of murine skin reactions poses similar challenges as those in a clinical setting, indicating a need for automated and standardized assessment techniques. The existing in-house mouse model relies on visual inspection in conjunction with a scoring table to grade the acute skin toxicity evident on mice daily over a 20-day period. This scoring process is reliant on trained staff for daily visual assessment, with corresponding inter-observer variation and lacks the capability for retrospective verification.

In recent years, there has been an increasing interest in developing automated methods for scoring to overcome the limitations associated with manual assessment (Medela et al. 2022; Guo et al. 2022). Automated scoring systems offer objective, reproducible, and efficient evaluation of skin reactions, enabling researchers to obtain quantitative data for analysis, comparison across different experimental conditions, and interinstitutional comparison.

In this study we present an imaging system for daily imaging of the mouse leg target in preclinical radiotherapy experiments, in combination with a deep learning-based image classification approach for objective skin toxicity classification. The classification system was trained on prospectively collected data from 120 mice and evaluated on a test set of 40 mice. In the final step, an observer study was conducted, where we tested the observers’ ability to classify toxicity from the imaging data and compared it to the automated system.

## Materials and methods

This section will present a deeper explanation of the animal model used in-house, the data acquisition setup, the dataset and the deep learning methods used on the data and, lastly the setup of the observer study conducted.

### Animal model and evaluation of radiation effects

The procedure for the radiation treatment has been described previously (Sørensen et al. 2022, 2015). All experiments comply to the ARRIVE guidelines(Percie du Sert et al. 2020) and institutional animal welfare regulations. As described in detail in Overgaard et al. (2023) irradiations were performed on the right hind leg using a Lucite jig for restraint in non-anesthetized animals, with the leg placed in a water bath maintained at 25 °C. Post-treatment, mice were returned to their cages for observation.

Skin damage assessment utilized a previously published scoring table (von der Maase 1984) as described in Overgaard et al. (2023); Sørensen et al. (2022). Scoring was performed manually in increments of 0.5, with a maximum score of 3.5. The scoring was based on the skin damage covering the whole foot and scorings were performed blinded to given treatment. Each mouse was scored daily from 8 to 28 days post-treatment. Included in the current study was data from irradiated mice, as part of ongoing radiobiological studies at our department, with doses from 20 to 58 Gy in order to obtain various levels of skin toxicity.

In accordance with previous pre-clinical trials (Sørensen et al. 2022, 2015), the outcome analysis exclusively consists of scores of 1.5 or higher. Therefore, the scores of 0.0, 0.5, and 1.0 were pooled together and not attempted stratified.

### Data acquisition and imaging setup

**Fig. 1:**
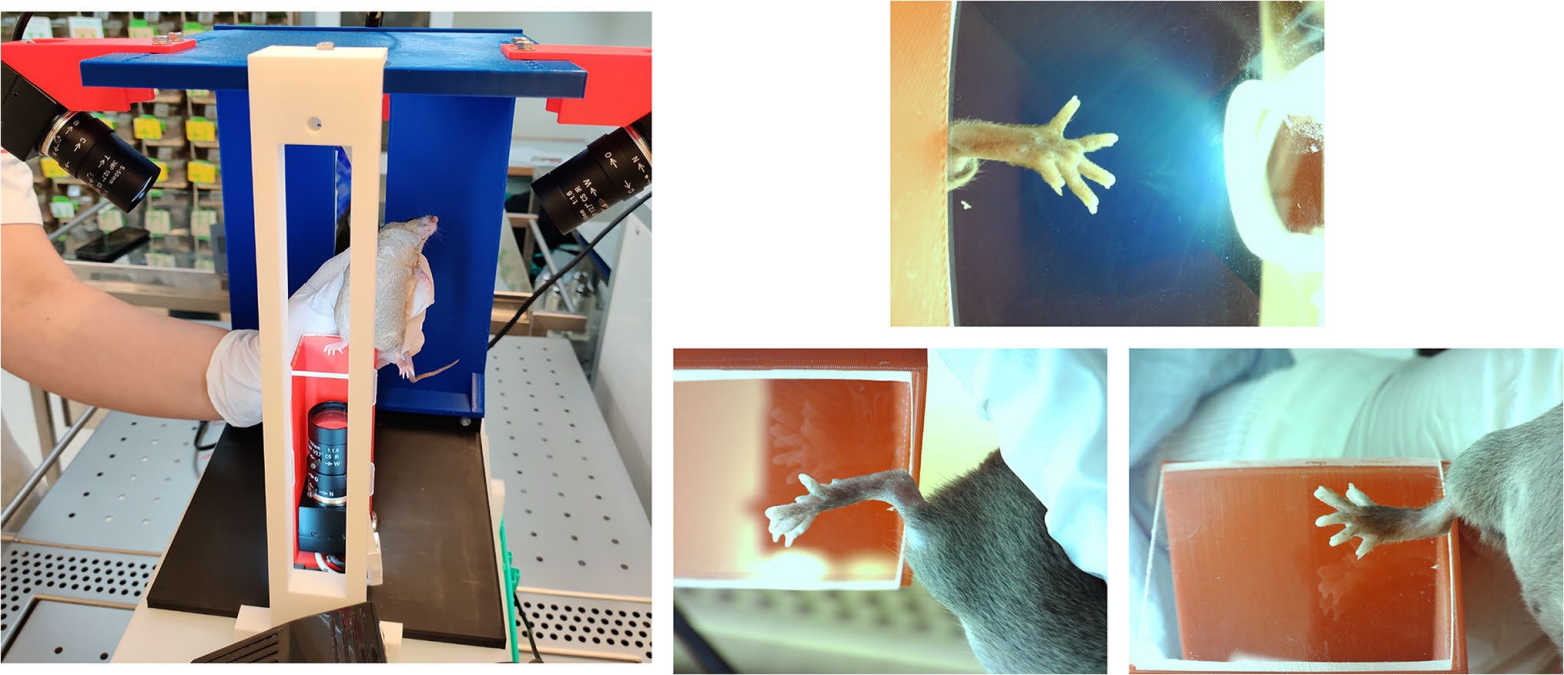
Left Panel: 3D printed apparatus for imaging the irradiated hind legs of the mice. Three camera setup, with one from the bottom and two from the sides. Right Panel: The three images, captured simultaneously from the setup

For the purpose of capturing standardized images of mouse hind legs, a dedicated apparatus was fabricated through 3D printing. This apparatus was outfitted with a trio of cameras, strategically positioned to capture comprehensive images of the irradiated hind leg of the mouse from various angles, ensuring the visibility of all facets of the foot. A depiction of this experimental arrangement is featured on Fig. 1.

Furthermore, in conjunction with the developed setup, a software application designed for mouse scoring was implemented, with a designated Graphical User Interface (GUI). This software makes the system store the images taken along with the toxicity score assigned by the assessor. The creation of this GUI was realized using the programming language Python (Van Rossum and Drake 2009), complemented by the PyQt5 package (Limited 2023). An additional implemented feature involved the presentation of captured images to the user, enabling identification of potential issues such as image blurriness or inadequacy. This mechanism enabled the user to initiate image recapture in instances where discernible flaws were observed.

### Dataset

The imaging setup was used during the skin toxicity assessment of four radiobiology trials, each comprised of 40 distinct mouse subjects. The trials were focused on determining dose response curves for acute and late toxicity in the context of proton / electron irradiation at conventional and ultra-high dose rates (FLASH), similar to Sørensen et al. (2022). Since this paper was only focused on the automated scoring system, no further details about the trials are deemed necessary. Each mouse subject was visually scored daily on acute skin toxicity at day 8–28 after irradiation by qualified assessors in accordance with the stipulated grading protocol, utilizing the previously delineated scoring table as a reference. The assigned manual score was used as the ground truth grade for model training. For every scoring instance, we also photographed the right hind leg using our photo apparatus. This resulted in the accumulation of 2514 mice scores with 7542 corresponding images (3 images per scoring instance). The different trials, with corresponding toxicity grades and applicable model phase can be seen on Table 1.

**Table 1:** Datatable of the four trials and their corresponding skin toxicity grades, that were collected over time. The percentage in the parentheses, is the percentage of the grade in the corresponding trial

Trial	H508	H510	H512	H513	All trials
Radiation type	proton	electron	electron	electron	
Mice subjects	40	40	40	40	160
Model phase	train/val	train/val	test	train/val	
Grade $<=$ 1	231 (45.8%)	346 (55.7%)	475 (65.2%)	446 (67.5%)	1498 (59.6%)
Grade 1.5	66 (13.1%)	137 (22.1%)	90 (12.4%)	76 (11.5%)	369 (14.7%)
Grade 2.0	28 (5.6%)	51 (8.2%)	53 (7.3%)	47 (7.1%)	179 (7.1%)
Grade 2.5	30 (6.0%)	38 (6.1%)	22 (3.0%)	14 (2.1%)	104 (4.1%)
Grade 3.0	86 (17.1%)	28 (4.5%)	38 (5.2%)	31 (4.7%)	183 (7.3%)
Grade 3.5	63 (12.5%)	21 (3.4%)	50 (6.9%)	47 (7.1%)	181 (7.2%)
Total	504	621	728	661	2514

As seen on Table 1, non of the trials contained 800 scoring instances, which is the expected amount if each mouse was scored each day for 20 days (20 scoring instances x 40 mice). This is due to various reasons such as the fact that when the mouse subject is on the highest toxicity grade, sometimes they did not get scored through the imaging apparatus to spare them the hassle and potential pain associated with the task.

### Deep learning study design

**Fig. 2:**
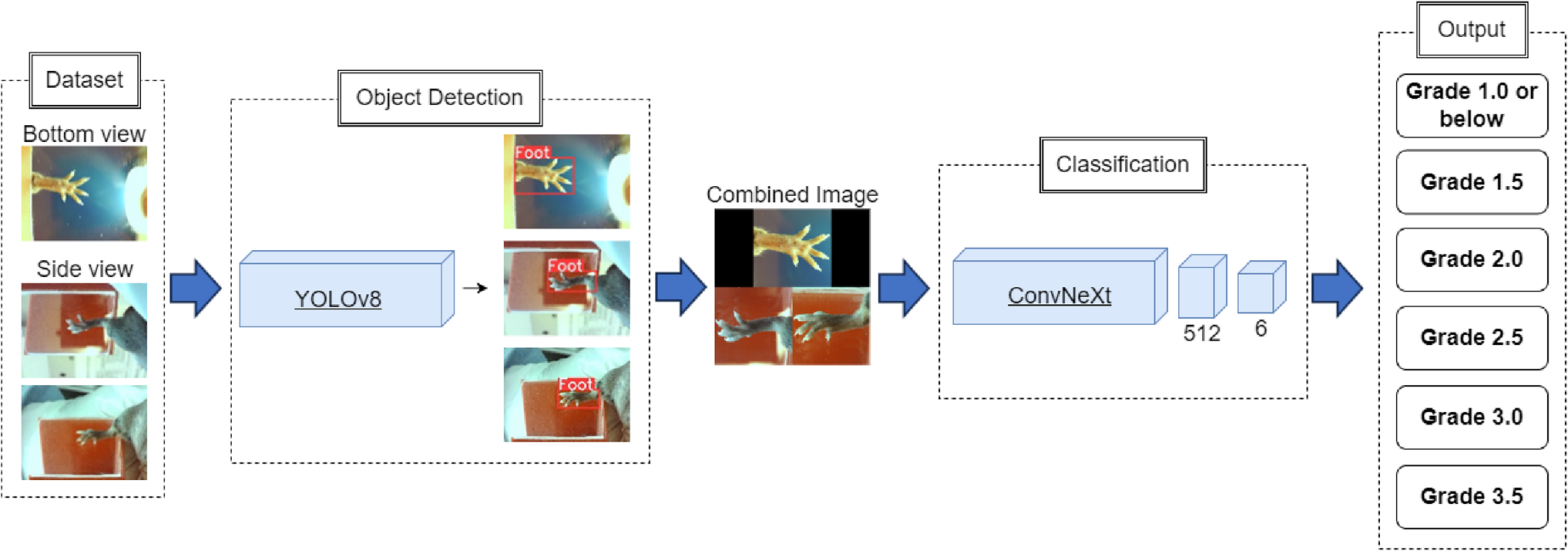
Schematic diagram of proposed framework for region of interest object detection and skin toxicity classification

The schematic diagram of the complete deep learning study design is shown in Fig. 2. The proposed deep learning (DL) framework designed for acute toxicity scoring is a two-step network, composed of object detection followed by classification.

The object detection integrates a DL network targeted at the designated region of interest (ROI), namely the radiated hind leg of the mouse. In this framework, the ROI was extracted utilizing YOLOv8 (Jocher et al. 2023). The ROI extraction from the three distinct images corresponding to a scoring instance, was finalized by the merging of these regions into a combined image. Subsequently, this combined image was directed through a modified ConvNeXt (Liu et al. 2022) DL classifier. The ultimate outcome of this process yielded one of the six classifications grades denoting skin toxicity severity.

According to the image orientation, images were divided into two subgroups: bottom view and top-side view. Within the bottom view subgroup, images were consistently positioned in the same arrangement within the final composite images. This strategic approach aimed to simplify the classification process for the model. In contrast, the two side view images, owing to their similarity, were designed to have interchangeable positions within the merged image and were used as a type of data augmentation.

### Automatic object detection of hind leg via YOLOv8

The ROI within the images corresponds to the radiated right hind leg of the mouse. For a subset of 1000 images (400 bottom view, 300 each top-side view) randomly selected from the data, manual delineation of these ROIs was performed. Subsequently, both the images and their corresponding bounding boxes were resized to 640x480 pixels, and then used as input for the YOLOv8 DL model (Jocher et al. 2023). The YOLOv8m configuration was employed in conjunction with the default hyperparameters during the training phase. The resulting output bounding box images from the model were subjected to a visual inspection, to optimize their value for the succeeding DL classifier. This inspection involved identifying cases of extreme blurriness or cases where the hind leg was too obscured and removing these cases from the dataset used by the DL classifier.

In addition to the delineated images, thirty background images devoid of mouse feet were introduced in the data, to mitigate the occurrence of false positives. Afterwards, the data were randomly partitioned into training (70%), validation (15%), and testing (15%) sets. The model was trained for a total of 200 epochs, adhering to the default hyperparameters prescribed by YOLOv8.

Model performance was evaluated by an assessment of its accuracy in effectively localizing the radiated hind leg within each image of the total acquired dataset, which encompassed a total of 7542 images. Each instance in which the object detection model identified a hind leg was considered a successful classification, while instances in which no ROI was detected were deemed unsuccessful classifications. All images with unsuccessful classification were visually inspected to determine the potential causes.

### DL network for skin toxicity classification

A convolutional network based on the ConvNeXt (Liu et al. 2022) was used for the skin toxicity classification. This network was developed for image classification tasks and is still considered state-of-the-art in this area. The ConvNeXt network was pretrained on the large public dataset ImageNet (Deng et al. 2009), which helps the model learn general visual features before being fine-tuned for this specific task. The main body of the model is the ConvNeXt-XL and an extra dense output layer has been implemented to reduce the number of neurons connected to the final output layer, which in turn is used as the classification layer.

#### Data balancing and augmentation

The dataset exhibited an imbalance concerning the distribution of images among different grades of skin toxicity severity as seen on Table 1. To address this disparity during the classification training, a balanced class weight scheme was employed within the classification network. The determination of these weights followed a calculation derived from the formula inspired by King and Zeng’s work (King and Zeng 2002). This formula was implemented in Python using the Scikit-learn package (Pedregosa et al. 2011). The weights were calculated as seen below:

$$\begin{aligned} \frac{n_{\text {total\_samples}}}{n_{\text {classes}} \cdot n_{\text {class\_counts}}} \end{aligned}$$

The generated weight tensor was then incorporated into the loss function during the classification training phase to address the data imbalance issue. To expand the training sample pool and improve classification robustness, data augmentation techniques were employed on the training data. The chosen augmentation scheme, namely RandAugment (Cubuk et al. 2019), utilizes 14 distinct augmentation operations, which can have different magnitudes. Therefore, the sole hyperparameters for this scheme are as follows:

**N**: Number of augmentation transformations to apply sequentially.
**M**: Magnitude for all the transformations.

These were respectively set to N = 2 and M = 9 during the training phase.

#### Network training

The network was trained, validated and tested on the trial basis outlined in Table 1. The table also provides details about the number of class instances involved in the study.

During training, the Lion (Chen et al. 2023) optimizer was employed in conjunction with class distance weighted cross-entropy loss (Polat et al. 2022). This specialized loss function, in contrast to standard cross-entropy, incorporates consideration of the distance between classes, imposing an additional penalty on the model when the predicted class deviates further from the ground truth. The optimizer’s hyperparameters were configured as follows: a learning rate of 5e-6, $\beta 1$ (first moment coefficient) set to 0.9, and $\beta 2$ (second moment coefficient) set to 0.99. No weight decay mechanism was integrated into the optimizer. The model trained for a total of 200 epochs.

To enhance the classification model’s robustness, a 5-fold cross-validation strategy was employed. This approach facilitated the utilization of the entire available training and validation subset. Furthermore, robustness was increased by leveraging all five models trained with the cross-validation as assessors for the final output. This mechanism allowed for the identification of potential discrepancies in predictions among the models. In instances where the five models’ predictions did not align, flags were raised, thereby introducing an element of robustness into the prediction process. The selection of models from the cross-validation was based on highest validation accuracy throughout the training, ensuring the inclusion of the most reliable models in the ensemble.

To conduct a quantitative assessment of the ensemble’s predictive performance on the test set, specific evaluation metrics were employed. These are the confusion matrix and overall accuracy of the model, along with class specific precision, recall and F1-score.

### Observer study

One of the goals of this study was to enable retrospective analysis of toxicity scores. Where the ground truth scores are based on direct visual inspection of the mouse leg, the intended setup for retrospective scoring relies on the imaging data. In the observer study we investigated the observer variability when using only the imaging data for scoring and used the manually assigned scores as ground truth reference.

The observer study involved five participants, all possessing expertise in grading skin toxicity severity via live visual inspection. Each observer was tasked with assessing 80 scoring instances, consisting of 10 instances for each toxicity grade, from 0.0 to 3.5. The scores 1.0 or below were afterwards combined. Observers were not provided with prior knowledge regarding the distribution of these grades within the study. The evaluation of inter-observer agreement was conducted using the Krippendorff’s alpha coefficient(Hayes and krippendorff 2007), chosen for its inherent suitability for assessing ordinal data, as is the case with toxicity grading. Krippendorff’s alpha value usually varies between 1.0 and 0.0, where 1.0 shows perfect reliability and 0.0 shows no reliability. According to Hayes and Krippendorff (Hayes and krippendorff 2007), alpha values above 0.8 indicate good reliability, 0.67$-$0.8 low reliability and <0.67 very low reliability. Additionally, the prediction output generated by the DL model was included as an "observer" to evaluate its inter-observer agreement in comparison to the human observers. The 80 scoring instances image set was therefore excluded from the DL training/validation/test sets.

In this study the Krippendorff’s alpha coefficient was calculated based on ordinal data and this calculation was subjected to 10.000 bootstrap iterations. Specifically, the Krippendorff’s alpha was computed for all conceivable pairs of observers, including the DL model considered as one of the observers.

The proficiency of correctly grading the toxicity severity was quantified using the accuracy of each observer. This was complemented by an average misclassification distance, which for the total amount of misclassifications per observer, calculated the average degree of misclassification from the original toxicity grade.

## Results

### Object detection performance

The ROI object detection model was applied to the entire dataset of images, allowing for the computation of accuracy for its object detection capabilities. Among the 7542 images in the dataset, the model successfully identified the hind leg in 7454 images, yielding an accuracy of 98.83%. The 88 images in which detection was unsuccessful could be categorized in the following subgroups:

1. Cases in which the mouse was entirely missing in the image: **39** instances
2. Scenarios involving obscured or extremely blurry hind legs: **38** instances
3. Instances where the model failed to detect the mouse leg: **11** instances

When we excluded the images in groups 1 and 2, the detection accuracy reached 99.85%.

### Classification performance

**Fig. 3:**
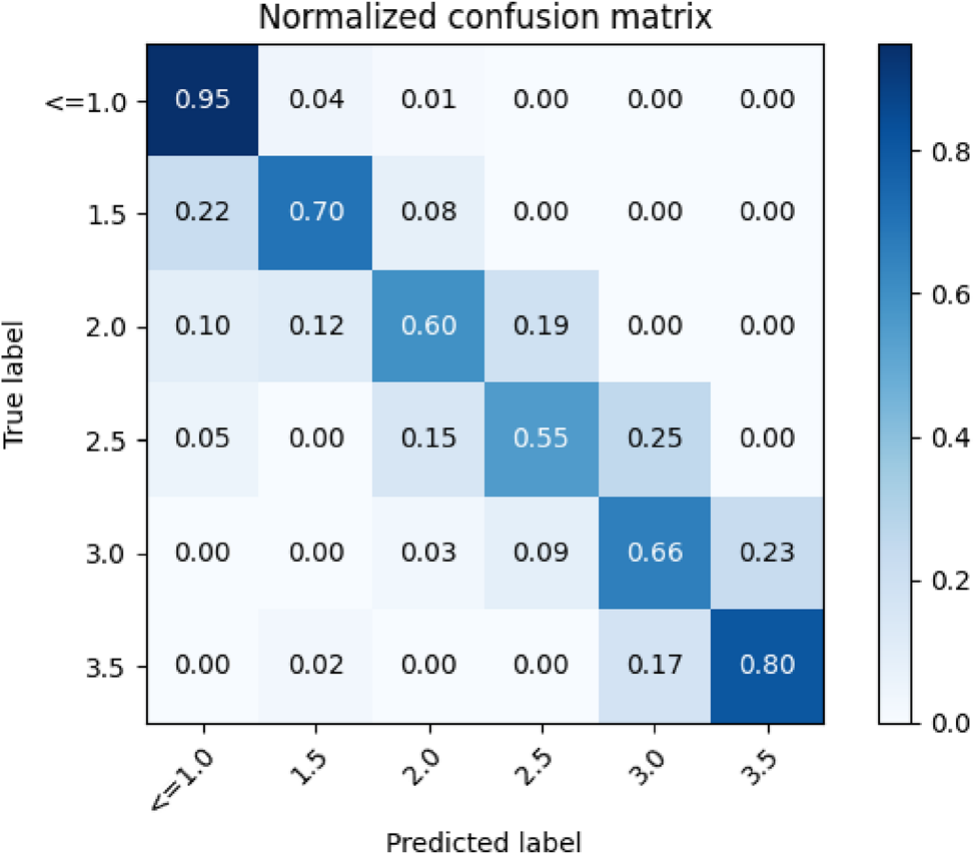
Normalized confusion matrix of the test set (Trial H512)

The classification was based on the 6 classes of skin toxicity severity and was evaluated on the H512 trial dataset Table 1. The dataset consisted of 655 scoring instances. The reason that it is not 728 instances, as shown on Table 1, is due to issues such as the object detection model was unsuccessful, images were deemed to blurry or obscured, or the data was used in the observer study instead. The total amount of the data used in the classification was 2375/2542 (93.43%) scoring instances. The overall testing accuracy was 85.34%. The corresponding normalized confusion matrix is presented in Fig. 3.

The results reveal a mere 13 instances where the classification deviated by more than one class, constituting 1.98% of all instances. In fact, only 3 instances exhibited errors exceeding two classes, all of which were subsequently identified as having been inaccurately labelled. Additional quantitative evaluations of the classification models testing performance are shown in Table 2 based on precision, recall, and F1-score.

**Table 2:** Evaluation metrics of skin toxicity severity classification for the test set (Trial H512)

Class	Precision	Recall	F1-score
Grade $<=$ 1.0	0.95	0.95	0.95
Grade 1.5	0.69	0.70	0.70
Grade 2.0	0.69	0.60	0.64
Grade 2.5	0.46	0.55	0.50
Grade 3.0	0.66	0.66	0.66
Grade 3.5	0.79	0.80	0.80

The results suggest that the model encounters challenges in certain toxicity grades, a phenomenon likely attributed to the limited sample space. Upon examination of the quantity of scoring instances, as presented in Table 1, a notable correlation emerges between the number of instances and the F1-score, indicating a dependency on data availability. Despite the seemingly low scores, it is crucial to note that when considering a grading accuracy that accounts for classes deviating by only one from the ground truth, all classes achieve an accuracy of 90% or higher.

**Fig. 4:**
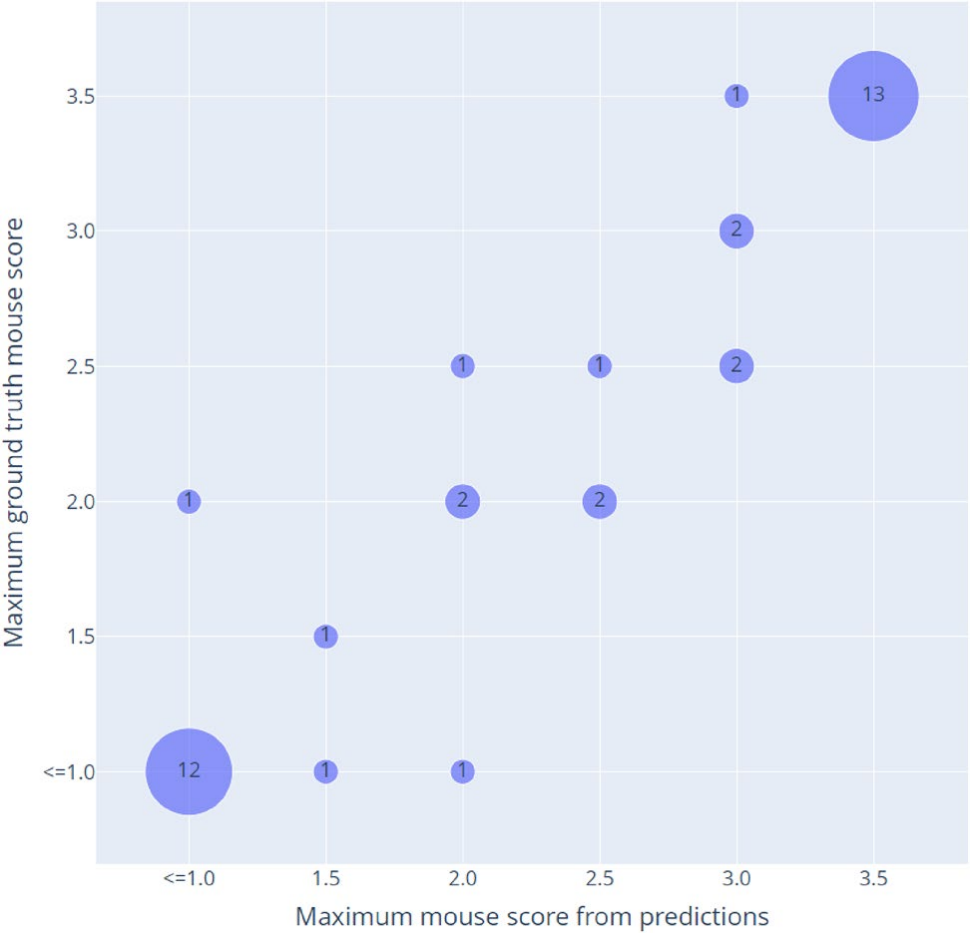
Maximum mouse score vs maximum predicted mouse score over the observation period for the test set (Trial H512). The numbers in the bubbles indicate the amount of instances in the data

As the preclinical trials utilize the maximum grading value of the mice observed over the entire trial period of 20 days(Sørensen et al. 2022) the performance of the DL model was evaluated based on this criterion as well. The results are illustrated in Fig. 4.

The findings reveal that the DL model accurately predicted the maximum toxicity score over the observation period in 31/40 (77.50%) of cases. In 7/40 (17.50%) of instances, it deviated by only a single grade from the ground truth, leaving 2/40 (5.00%) where the deviation reaches two grades.

### Observer study performance

In the observer study, the mean alpha value for each observer, along with the corresponding mean 95% lower and upper confidence intervals, were derived along with a comparison between ground truth score and the grade derived from the image inspection. A summary of these statistics is shown on Table 3.

**Table 3:** Mean Krippendorff’s alpha value and mean 95% lower confidence interval (LCI) and upper confidence interval (UCI) for each observer, based on their pairwise calculation with all possible observer pairs. Along with accuracy, average misclassification distance (AMD) and accuracy within 1 class deviation for each observer

Metric	Alpha (95% LCI - UCI)	Accuracy	AMD	Accuracy (within 1 class)
Observer 1	0.9038 (0.8632$-$0.9390)	66.25%	0.6667	90.00%
Observer 2	0.9146 (0.8718$-$0.9492)	**72.50%**	0.6818	92.50%
Observer 3	0.9178 (**0**.**8782** $-$0.9514)	60.00%	0.6094	91.25%
Observer 4	0.8878 (0.8410$-$0.9298)	61.25%	0.5806	93.75%
Observer 5	0.8900 (0.8416$-$0.9312)	68.75%	0.6200	93.75%
Model	**0**.**9184** (0.8758-**0**.**9538**)	71.25%	**0**.**5652**	**96.25%**

Bold text indicates the best value for each column

The results indicate that the observers all have alpha’s above 0.8 indicating a high agreement, and the DL model has a higher mean pairwise alpha value than any of the human observers, but as the 95% confidence interval all overlap there is no significant differences between observers. For further results, a Krippendorff’s alpha matrix with each pairwise observer value can be found in the supplementary section.

The ground truth comparison results indicate that the DL model achieved a higher accuracy than four out of five human observers and has the lowest average misclassification distance among all the observers. Also, for the accuracy within one class distance the DL model showed best performance.

## Discussion

The presented study introduces an innovative imaging system integrated with a deep learning-based classification model for objective skin toxicity assessment in preclinical radiotherapy trials. We have shown that it was feasible to use the imaging setup on a daily basis, where 2375/2542 (93.43%) of the acquired images were used. We have also shown that the detection of the right hind leg in the images was reliable (99.85%), and have shown a classification accuracy of 85.34%. Finally, in the observer study the DL model was shown to perform on par or better than the human observers.

The object detection model achieved high accuracy in identifying hind legs, demonstrating robust performance even in challenging scenarios such as obscured or blurry images. If we only focused on the reasonable errors in mouse leg detections, the model achieved a success rate of 99.85%. This was expected as YOLOv8 has shown good performance in other medical segmentation tasks (Nejad et al. 2023; Nakarmi et al. 2023).

The subsequent classification model exhibited an overall accuracy on the test set of 85.34% and on the observer study set of 71.25%. This accuracy is not perfect, but considered high, and most errors are only a single class off. As a comparison the average accuracy of the human observers in the observer study is 65.75%. As there are expected to be observer variations in the ground truth scores, the errors with a single class off, are basically impossible to eliminate, and can be accepted as long as it consists of a minority of instances. It is noteworthy that the DL model has a lower accuracy on the observer study set than on the test set. A likely explanation is the selection bias in the observer study, making the classes balanced, which is not the case in the test set, however taking this bias into account the accuracy was still expected to be higher. The performance evaluation, including precision, recall, and F1-score, suggests that the model encounters challenges in certain toxicity grades, potentially linked to data availability. These discrepancies are expected to vanish as more data is gathered, and the model is retrained.

Moreover, the system’s ability to capture the maximum toxicity score over the observation period aligns with preclinical trial objectives. The findings underscore the model’s proficiency, accurately predicting the maximum score in the majority of cases and exhibiting only minor deviations in a small percentage of instances. The two instances where the model was deviating two classes from the ground truth score were subjected to further analysis with one of the qualified assessors, and both instances displayed similar characteristics. One was predicted as a 1 or below, and the ground truth score was 2. The ground truth score had a spike in the scoring as function of time and was only above 1 or below a single day, and looking at the captured images, the assessor also expected the score to be 1 or below from the images. The second was predicted as a 2, but the ground truth score was 1 or below. Here the predicted score indicated a spike in the scoring were the prediction rose to 2 and stayed there for another day, while the ground truth score was 1 or below through the scoring period. On these images the mouse toes were captured while clumping tightly together, which is often seen as a feature on scores of 2 or above. This indicates one of the issues with the image scoring, which can be fixed by manually assuring that the mouse spread out its’ toes if possible.

Through the flagging process with the five models and their internal agreements, it is possible to flag the discrepancies in their agreements and enable even further robustness in the setup. The result from this process can be found in the supplementary section.

The observer study revealed a high inter-observer agreement among human observers and the DL model, along with a respectable accuracy. This indicates that the images can be used for retrospective verification. The DL model surpassed the individual human observers’ pairwise agreements, and consistently outperformed most human observers in accuracy and average misclassification distance, emphasizing its potential as a reliable and consistent evaluator. The scores in the observer study are based on the images only, and the observer variation is therefore also only based on this. However, the ground truth scores are also expected to have observer variation, but unfortunately, we do not know the extent of it.

The system shows promising results in addressing the limitations of a manual scoring system, however there is still fine-tuning left for it to be a distributable finalized system. The intended final system would follow this proposed setup: Direct processing of the data, meaning leg detection and classification, and propose the toxicity score to the user. From the results, we can expect this to be correct in about 85% of the cases (This varies in the different classes and has the possibility to be improved through the flagging system.) In case the operator disagrees with the score, this can be corrected immediately. This will also provide an opportunity for finetuning the classification model, as the instances with disagreement can be used in a subsequent retraining.

As the training time of new staff for the daily visual inspection takes time, this system could be used instead for the scoring. However, as some classes still have a relatively low accuracy, the automatic method, with possibility for manual intervention is proposed. As the system improves the need for the manual intervention is expected to become nearly non-existing.

## Conclusion

The study successfully addresses the limitations of manual scoring systems, offering an automated, objective, and reproducible approach to acute skin toxicity assessment. The results demonstrate the potential of integrating imaging and deep learning in preclinical research, providing a valuable tool for researchers and clinicians in evaluating skin reactions in radiation therapy. The developed system not only enhances accuracy but also enables retrospective toxicity scoring, addressing a critical gap in current methodologies. Future work could focus on expanding the model’s training dataset and refining the system for the proposed setup mentioned.

## Supplementary Information

Below is the link to the electronic supplementary material.

Supplementary file 1 (docx 168 KB)
