# Supplementary material for: Automated acute skin toxicity scoring in a mouse model through deep learning
Source: Radiat Environ Biophys. 2024 Nov 6;64(1):45–53. doi: 10.1007/s00411-024-01096-x (PMC11971058; doi:10.1007/s00411-024-01096-x)
Supplement: Supplementary file 1 — (docx 168 KB) [file 411_2024_1096_MOESM1_ESM.docx]

**Supplementary Information (SI)**

**Article title:** Automated acute skin toxicity scoring in a mouse model through deep learning

**Journal name:** Radiation and Environmental Biophysics

**Author names:** Morten Sahlertz, Line Kristensen, Brita Singers Sørensen, Per Rugaard Poulsen, Folefac Charlemagne Asonganyi, Priyanshu Sinha, Jasper Nijkamp

**Affiliation and e-mail address of the corresponding author Morten Sahlertz**

Danish Centre for Particle Therapy, Aarhus University Hospital, Aarhus, Denmark

Email: mortsa@clin.au.dk

Phone: +45 29708960


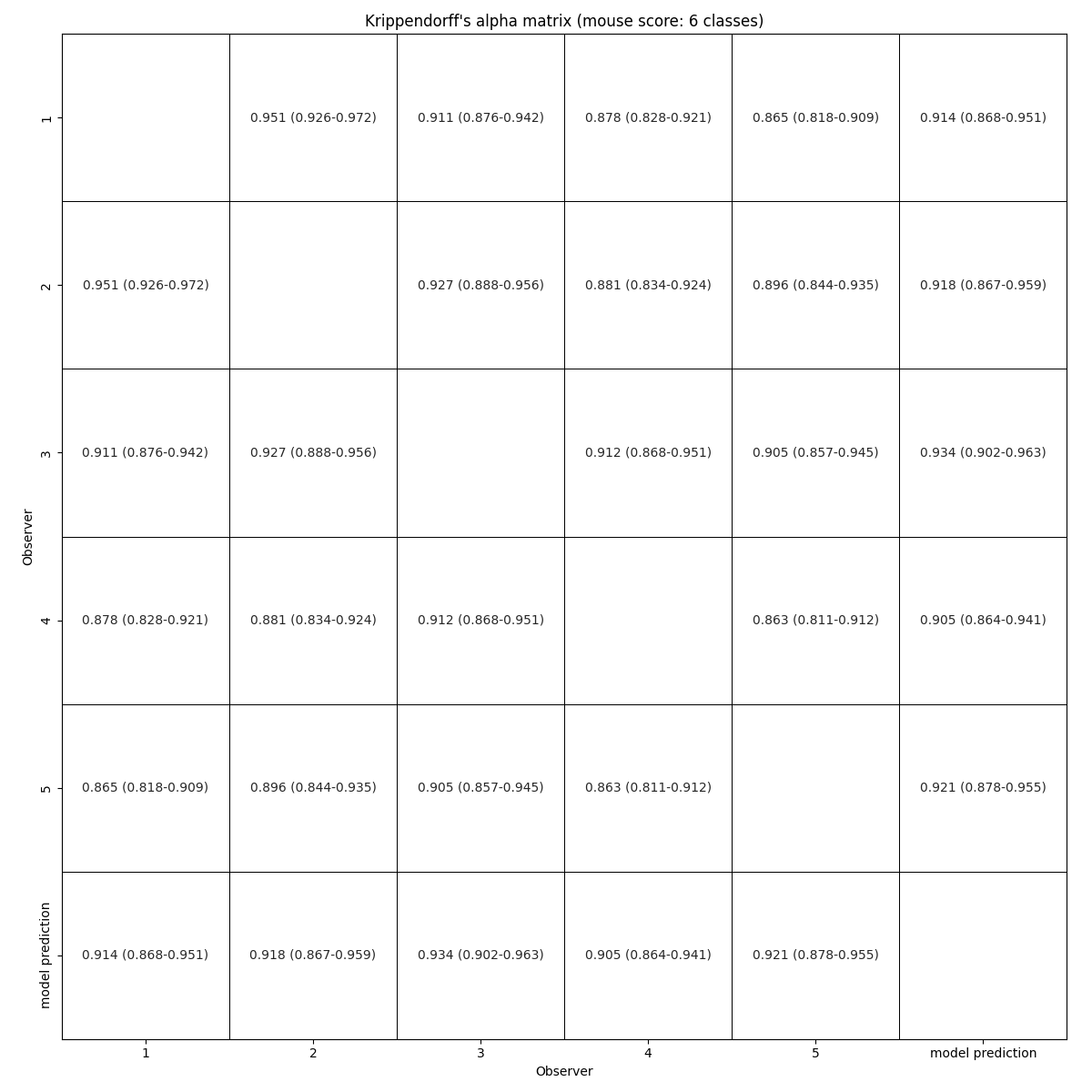


Figure S1 – Krippendorff’s alpha matrix with each pairwise observer value. The value in each square corresponds to the calculated alpha value with the lower 95% lower to upper confidence intervals in the parentheses, for the chosen observer pair.


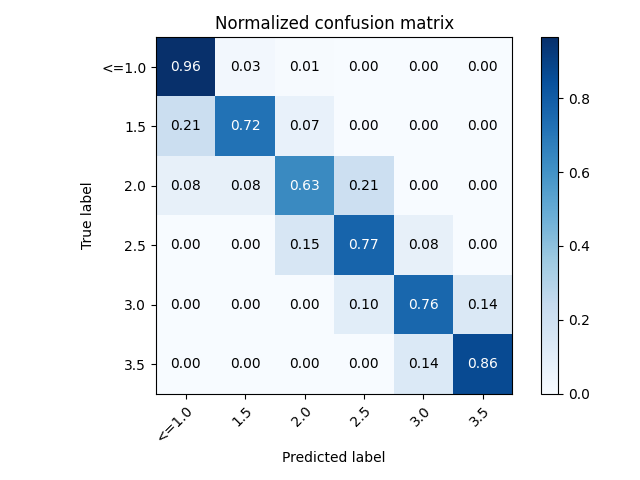


Figure S2 – Normalized confusion matrix for the test set (Trial H512), where 4 of 5 of the models agrees on the prediction. This occurred 608/655 (92.82%) times in the test set and reached an accuracy of 89.47%. The 47 instances with less agreement from the models had an accuracy of 16/47 (34.04%) and would be flagged if encountered in the system.


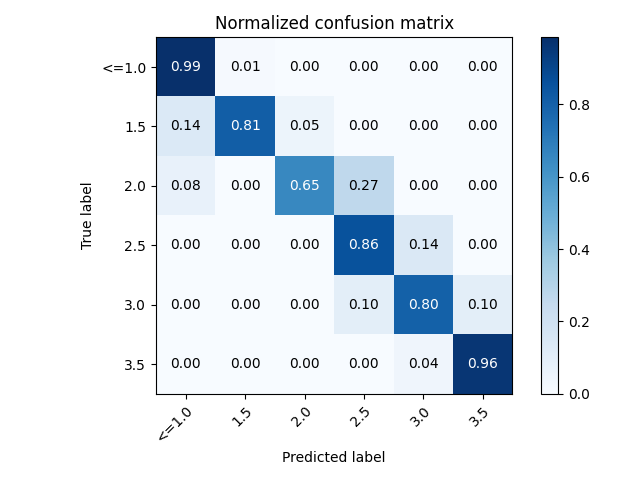


Figure S3 - Normalized confusion matrix for the test set (Trial H512), where 5 of 5 of the models agrees on the prediction. This occurred 538/655 (82.14%) times in the test set and reached an accuracy of 94.05%. The 117 instances with less agreement from the models had an accuracy of 54/117 (46.15%) and would be flagged if encountered in the system.
